# Strengthening immunity through healthy lifestyle practices: Recommendations for lifestyle interventions in the management of COVID‐19

**DOI:** 10.1002/lim2.7

**Published:** 2020-10-03

**Authors:** Ifeoma Monye, Abiodun Bamidele Adelowo

**Affiliations:** ^1^ Department of Family Medicine National Hospital Abuja Nigeria; ^2^ Brookfield Centre for Lifestyle Medicine Abuja Nigeria; ^3^ Department of Medical Services State House Medical Centre Abuja Nigeria; ^4^ Department of Human Kinetics and Health Education University of Lagos Morpeth Nigeria

**Keywords:** COVID‐19, healthy lifestyle practices, immune system, lifestyle intervention, SARS‐CoV‐2, supportive care, viral infections

## Abstract

Although the practice of strengthening the immune system may not guarantee that people will not contract severe acute respiratory syndrome coronavirus 2 (SARS‐CoV‐2), there is evidence that suggests that the likelihood and severity of many viral infections can be considerably reduced if appropriate measures are taken to increase the efficacy of the immune response to invading organisms. Evidence‐based public health measures to reduce viral spread include—personal isolation, physical distancing, wearing facial masks, frequent hand washing with soap and running water, not touching the face, vaccination, etc. However once infected, recovery relies on host immunity to eradicate the foreign invaders, with symptomatic management, which is the core management strategy in COVID‐19 management guidelines. Researchers have observed that severe COVID‐19 prevalence and mortality is highest in people with preexisting conditions. Eighty percent of these preexisting diseases are lifestyle related. Also, the science of strengthening the immune system by adopting appropriate lifestyle changes is still evolving, especially among the suspected and confirmed COVID‐19 cases. This article briefly highlights the immune response to viral infections including COVID‐19 and refers to evidence that healthy lifestyle practices, enshrined as core aspects of lifestyle medicine, can strengthen the immune response to infections. This may improve outcome in COVID‐19. We therefore recommend specific evidence‐based lifestyle intervention measures that should be considered in the management of COVID‐19.

## 1 INTRODUCTION

The world completed the dusk of 2019 and the dawn of 2020 with a new public health challenge. A pneumonia of unknown cause with devastating and rapid fatalities, detected in Wuhan, China, was first reported to the World Health Organisation on December 31, 2019.^1^ A new virus, SARS‐CoV‐2, was identified as the cause of a new viral disease, coronavirus disease 2019 (COVID‐19), that has ravaged the global population.^2^ Since the outbreak of the disease, there is no approved vaccine yet or guarantee one is possible although phase I studies are promising.^3^ There is now evidence that antivirals may work^4^ and that dexamethasone improves mortality for patients on oxygen and intensive care.^5^ But general supportive care and symptomatic management remain core recommendations, as evidence rapidly evolves.^2^, ^6^ The present supportive care for COVID‐19 patients is likely to be more effective in achieving its desired goal for full recovery, if it is composed of evidence‐based comprehensive measures that have the potential of optimizing the resilience of the immune system of the affected individuals.

The clinical presentation of COVID‐19 can be grossly divided into three stages: stage I, an asymptomatic incubation period with or without detectable virus; stage II, nonsevere symptomatic period with the presence of virus; and stage III, severe respiratory symptomatic stage with high viral load.^7^ Available statistics suggest that majority of COVID‐19 patients enter only stage I or II before achieving viral remission. The data so far would suggest that about 85% of patients will make full recovery from COVID‐19 without significant symptoms.^7^ A major strategy in the present clinical management of COVID‐19 is to prevent the progression of the disease from stage I to III.^7^ Even though researchers and clinicians are still studying all the factors that may contribute to the progression of COVID‐19 from one stage to another, helpful insights obtained from emerging genetic and clinical evidence suggest a similar clinical path to fairly well‐studied coronaviruses like the severe acute respiratory syndrome (SARS) virus and the Middle East respiratory syndrome (MERS) virus.^8^ Information from the study of these other coronaviruses suggest that the host immune system plays a major and critical role in the disease progression and outcome of these viruses.^8^ Therefore, an effective immune response might also be critical to the successful contraction and progression of COVID‐19. A poor immune response has been postulated as one of the reasons why the disease seems to be more severe in the elderly, the immunocompromised, or in people with comorbid chronic diseases. It therefore can be argued that any supportive care that has the potential of strengthening the innate and adaptive immunity of the host will likely have significant positive influence on the disease progression and prognosis of COVID‐19.

## 2 A BRIEF INSIGHT TO THE GENERAL IMMUNE RESPONSE TO VIRAL INFECTIONS

The body responds to almost all viral infections by the recruitment and activation of certain inflammatory cell types like macrophages and in some cases neutrophils. This in turn leads to the release of a range of proinflammatory and tissue‐damaging molecules like cytotoxic cytokines, cationic proteins, lipid mediators, metalloproteinases, and components of the oxygen burst.^9^ The extent and severity of the resulting tissue damage is usually modified by the innate and adaptive immune system; a critical process that eventually determines the clinical outcome of the infection.^9^ Specifically, the host innate immune system counters the negative effect of viral infections by producing anti‐inflammatory cytokines (such as IL‐10 and TGF‐β) and mediators (such as resolvins and galectins). These primarily block proinflammatory cytokines and chemokine production, Major Histocompatibility Complex class II expression, also interfere with many signaling pathways that result in proinflammatory cytokine production.^9^ Interferon gamma (IFN‐α) is also produced to activate macrophages and natural killer/cytotoxic cells (NK cells), which destroy the virus and the infected cells, respectively.^10^

The adaptive immunity against viral antigens is set in motion with the activation of cytotoxic T lymphocytes—CD8+ cells that exert cytotoxicity by lysing the infected cells and viruses. The adaptive immune system also activates CD4+ cells, which collaborate with B cells to produce antibodies.^10^ The antibodies assist by binding to the viruses and prevent them from penetrating a noninfected cell. The antibodies play a fundamental protective role against the reinfection of some viruses in a previously sensitized host (whether by a prior infection or immunization), by intercepting the process of viral‐host cell binding.^10^ Although other factors—like viral dose, route of infection, age of the host, host genetic susceptibility, concurrent infection with other agents, or past exposure to cross‐reactive agents—also play important roles in host‐virus interplay, the effective immune response of the host is particularly critical in determining the susceptibility, duration, severity, and ultimately the clinical outcome of a viral infection.^9^, ^10^

## 3 SPECIFIC IMMUNE RESPONSE TO COVID‐19

Early work is already being done, and knowledge is growing exponentially on the specific immune response to COVID‐19. What is known for now is that the virulence and pathogenicity associated with SARS coronaviruses develop as a result of viral activation of the cytoplasmic NLRP3 inflammasome within activated macrophages and Th1 immune cells. In turn, this inflammasome releases proinflammatory cytokines, like IL‐1B and IL‐18, which dictate the pathogenic inflammation responsible for the virulence and symptomatology of SARS‐CoV‐2.^11^

**FIGURE 1:**
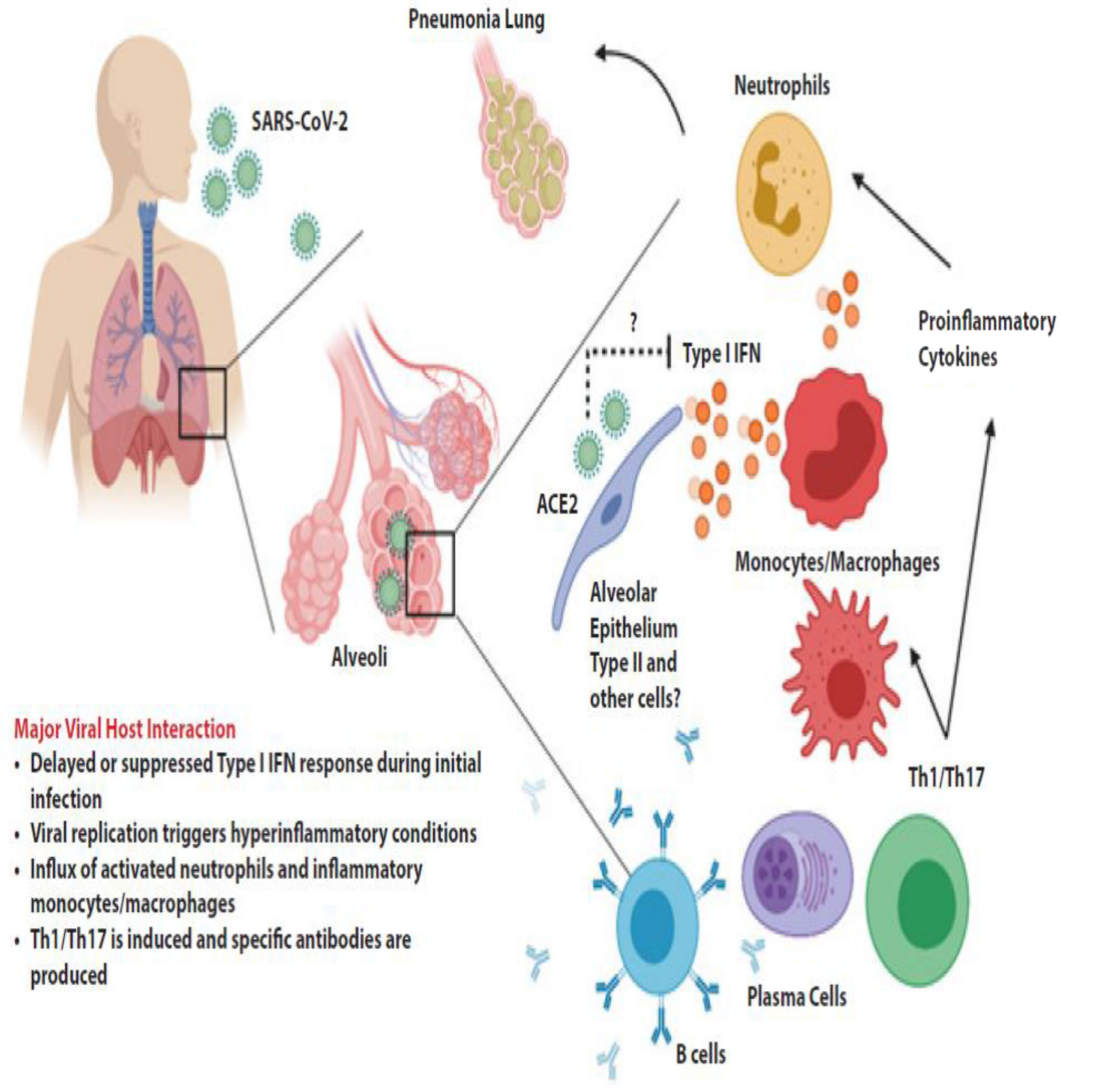
Proposed host immune responses during SARS‐CoV‐2 infection. This figure was adapted with permission from Prompetchara et al^8^

Studies on infected patients also support the claims that the host immune response to COVID‐19 is similar to that of other coronaviruses like SARS‐CoV‐1 and MERS‐CoV (see Figure 1).^8^ A study that investigated confirmed COVID‐19 patients in China reveals that the body responded by increasing the total neutrophils (38%), serum IL‐6 (52%), and C‐reactive protein (CRP; 84%), but reduced the total lymphocytes (35%). Another study that was conducted on intensive care unit (ICU) COVID‐19 patients in China similarly revealed an increased total neutrophil and decreased total lymphocytes, which correlate with disease severity and death. Furthermore, the patients had higher plasma levels of many innate cytokines, IP‐10, MCP‐1, MIP‐1A, and TNF‐α.^8^ Reports on intensive care audits for United Kingdom COVID‐19 patients also showed that preinfection well‐being is a critical factor in determining host susceptibility to severe cases of COVID‐19.^12^

Other studies on SARS‐CoV‐1 and MERS‐CoV, which are both coronaviruses of similar genetic composition as SARS‐CoV‐2, suggested that delayed and weak adaptive antibody response are associated with severe clinical outcome.^8^ Yet another study that was done in an in vitro plaque assay demonstrated that the sera (containing antibodies) of COVID‐19 patients were effective in neutralizing all the SARS‐CoV‐2 viruses in the assay.^8^ Some other data also suggest that there are mild or severe cytokine storms in patients with severe COVID‐19, which is also an important cause of death.^13^ Studies have also demonstrated that the plasma level of C‐CRP positively correlated with extent of lung lesions and reflect severity of COVID‐19.^14^, ^15^

Apart from the direct role that adverse immune response plays in COVID‐19, research has shown that the immune system plays a central role as well, in many of the underlying chronic diseases.^16^ Inflammation resulting from immunologic maladaptation appears to be the central driving force in the pathologic processes in most of these chronic degenerative diseases.^16^ This inflammatory process tends to affect nearly all organ systems of the body including the skin, endocrine glands, gut, lungs, kidneys, and musculoskeletal and cardiovascular system.^16^ In the long term such immunologic maladaptation may lead to chronic disease states like atherosclerosis, hypertension, cancer, epilepsy, Alzheimer's disease, Parkinson's disease, multiple sclerosis, and liver cirrhosis.^16^ Consequently, the proinflammatory process that characterizes COVID‐19 may worsen the underlying inflammatory process of most of these comorbid chronic diseases.

These clinical findings suggest that the disease progression and severity of COVID‐19, as well as the possible underlying chronic diseases, are likely determined by highly proinflammatory cytokines and processes. Thus, strengthening the innate and adaptive anti‐inflammatory processes of the immune system is a critical step, as this plays an important role in countering the proinflammatory process and in positively modifying the disease outcome in the suspected and nonsevere stages of COVID‐19.^8^ Therefore, boosting the host immune system should be a key management strategy of the disease.^10^ However, it is worth noting that a strong immune system may not be advantageous for stage III COVID‐19 cases.^10^ The treatment of cytokine storm and other appropriate forms of clinical management should be considered priority at this stage.^13^

## 4 THERAPEUTIC LIFESTYLE INTERVENTIONS: AN EVIDENCE‐BASED SUPPORTIVE CARE STRATEGY

The science correlating healthy lifestyle and enhanced immune function is still evolving, with consistent causative relationships between lifestyle modification and immune system improvement yet to be elucidated.^17^, ^18^ Also, there exists limited published information on the possible clinical benefits of optimizing the lifestyle of COVID‐19 patients, yet available evidence suggests that healthy lifestyle practices among patients with infectious diseases, especially those of viral origin, may boost their immune system and shorten the duration of their disease. Evidence also exists which suggest that some components of unhealthy lifestyle (like poor diet, physical inactivity, stress, smoking, alcohol, loneliness, and poor sleep) may significantly impair the immune system and predispose people to greater susceptibility to infectious diseases.^18^

Furthermore, the practice of healthy lifestyle is regarded by many clinicians as an important first‐line measure to strengthen the immune system either in the prevention of, or treatment of, a particular infectious disease.^18^ Healthy lifestyle has also been identified as the single most important step that an individual can adopt in order to naturally keep the immunity strong and healthy.^18^ This is so because the systems of the human body, including the immune system, function more effectively when they work in harmony and balance, protected from environmental assaults and bolstered by healthy lifestyle choices.^18^, ^19^ For instance, early research reports suggest that obesity (a condition that places a lot of strain on the immune system as well as other systems of the body) could double the chances of admission in hospital and worsen the outcome for COVID‐19.^20^, ^21^ Prime Minister Boris Johnson was reported to have attributed his serious disease, requiring intensive care treatment, to his weight and higher body mass index (BMI).^20^ Evidence from COVID‐19 studies so far shows that unhealthy lifestyle practices, such as poor nutrition, smoking, obesity and associated diseases, are linked to poor outcomes.^18^

Aside the immune strengthening potential, available information also suggests that healthy lifestyle practices may also play a significant role in the overall well‐being of the patients. Taking a cue from preceding epidemics from diseases of genetic similarity to COVID‐19, mainly the SARS and MERS, in addition to the primary dysfunction of the respiratory system, there may also exist extrapulmonary manifestations from the coronavidae family.^22^ For instance, acute and chronic cardiovascular presentations and complications are possible clinical outcomes of most coronaviruses.^22^ Preexisting cardiovascular disease may also worsen, contributing to adverse clinical outcomes.^22^ Other comorbid diseases, like diabetes mellitus, hypertension, obesity, asthma, chronic obstructive pulmonary disease, and cancers, may also be indirectly negatively affected by coronaviruses.^21^, ^23^ For these reasons, the ideal clinical management guideline of coronaviruses, like COVID‐19, should comprise comprehensive lifestyle changes that seek to optimize not only the immune system but also the overall health and well‐being of the patients. Some of the scientifically agreed lifestyle measures that may strengthen the immunity and ensure better general well‐being either singly or in combination are presented below.

### 4.1 Nutrition

There appears to be an association between nutrition and immunity (especially in the elderly), and this connection can be traced to diverse paths.^18^ First, the immune cells need adequate supply of energy (from foods) in order to effectively respond to invading infections.^24^ Second, an “activated” immune system further increases the demand for energy during periods of infection, with greater basal energy expenditure during fever, for example.^25^ Third, an adequate supply of amino acids is required for the production of quality immune proteins such as immunoglobulins, cytokines, and acute‐phase proteins. Fourth, availability of adequate amounts of minerals (like iron, zinc, and magnesium) is essential for the synthesis of nucleotide and nucleic acid of the immune cells, while vitamins (like vitamins C and E) are also needed in adequate quantity to support the antioxidant defense mechanism, which is needed to limit tissue damage in viral infection.^24^

A single micronutrient can exert multiple diverse immunological effects, such as in the case of vitamin E, where it has a multiple role as an antioxidant, inhibitor of protein kinase C activity, and potentially interacting with enzymes and transport proteins for effective immune response during an infection.^25^ The antioxidative effects of some vitamins (like vitamin C) may be particularly important during heavy viral infection when oxidative stress is likely to increase.^24^ Also, some micronutrients (like vitamins A and D), if available in adequate quantity, can directly regulate the gene expression of the immune cells and optimize their antiviral functions.^24^, ^26^

By comparison, consuming diets that are nutrient‐poor over a period of time, may eventually lead to a state of malnutrition, which has multiple direct negative effects on the immune system and may result in higher susceptibility to and severity of infections, like COVID‐19.^18^, ^27^ Furthermore, unhealthy diets (diets characterized by high sugar, trans and saturated fats content, but low in complex carbohydrates, fiber, micronutrients, and other bioactive molecules such as polyphenols and omega 3 polyunsaturated fatty acids), have been associated with the promotion of constant, low‐grade, systemic inflammation, caused by the maladaptation of the immune system and other cells (like adipocytes) to these types of food.^25^ A situation that may also weaken the immune response to infections like COVID‐19 and/or increase the severity of the disease.

Studies have further elucidated that the direct viral infection of the intestinal mucosa in COVID‐19 patients may lead to gastrointestinal symptoms such as abdominal pain, nausea, vomiting, and diarrhea. Researchers from the Hubrecht Institute in Utrecht, Rotterdam, Maastricht, the Netherlands, have found that the coronavirus SARS‐CoV‐2 can infect cells of the intestine and multiply there.^28^ The use of antiviral and anti‐infective drugs may also lead to gastrointestinal symptoms. This may result in distortion of the microecological balance of the intestine, manifesting as a significant reduction of the intestinal probiotics (such as lactobacillus and bifidobacterium). Intestinal microecological imbalance in these COVID‐19 patients may further lead to bacterial translocation and secondary infection.^29^ Therefore, in the management of COVID‐19 cases, it is essential to maintain the balance of the intestinal microecology as much as possible, primarily by adequate healthy nutritional support.^29^ Furthermore, unhealthy diets deplete prebiotics and probiotics, modify the gut microbiota and create a distortion in balance between both. This is another way that poor nutrition may affect immunity and modify the severity of infection. When nutrition is optimal, there is a good balance between prebiotics and probiotics.^24^ However, the bioavailability of most of the immune promoting nutrients is best optimized if the nutrients are consumed through healthy foods and not by dietary supplementation.^30^

Experts have postulated that the optimal nutrition for the best immunological outcomes to various infections would be that type of nutrition which has the potential to efficiently support the functions of the immune cells, allowing them to seamlessly initiate effective and rapid responses against pathogens while preventing any underlying chronic inflammation.^25^ Dietary patterns whose primary focus or emphasis is on the regular consumption of vegetables and fruits, like the whole food plant‐based diet and the Mediterranean diet, are best fit for this type of nutrition. These are diets that emphasizes the daily consumption of fresh vegetables, fruits, beans, lentils, whole grains, nuts, seeds, fish, and “healthy” dietary fats, while avoiding sugar, unhealthy fats, and salt significantly.^25^, ^31^, ^32^, ^33^ These types of diet are very effective in lowering inflammation, as they are fiber‐rich, nutrient‐dense, antioxidant‐rich, and very low in proinflammatory foods (like trans fats and sugar).^25^, ^32^, ^33^, ^34^ Compared to other types of diets, they are also rich in flavonoids—which have been found in vitro to reduce NLRP3 inflammasome signaling and consequently reduce the expression of proinflammatory cells like NF‐kB, TNF‐a, IL‐6, IL‐1B, and IL‐18.^11^

### 4.2 Physical activity

There is clear evidence that the immune system responds to regular physical activity, with the extent and duration reflecting the degree of physiological stress imposed by the workload.^34^, ^35^ Available evidence suggests that during moderate‐ and vigorous‐intensity aerobic exercise of less than 60 min duration, there is an activation of the antipathogen activity of tissue macrophages together with enhanced recirculation of immunoglobulins, anti‐inflammatory cytokines, neutrophils, NK cells, cytotoxic T cells, CD8+ T lymphocytes, and immature B cells, all of which play critical roles in immune defense activity and healthy metabolic health.^35^ Over time, if done on most days of the week, the summative effect of these transient exercise‐induced immune activities will lead to enhanced immune surveillance against pathogens and lower systemic inflammation—an important process in the prevention and treatment of infections, including those of viral origin.^35^ Several randomized clinical trials and epidemiologic studies have consistently supported this inverse relationship between moderate‐intensity exercise and incidence of infection, especially upper respiratory tract infections (URTI).^35^ Metabolically, moderate exercise induces acute elevations in IL‐6, a process that has direct anti‐inflammatory effects, and improves glucose and lipid metabolism over time.^35^

Other anti‐inflammatory benefits of regular moderate‐intensity physical activity/exercise may include—general improvement in the control of inflammatory signaling pathways, release of muscle myokines that stimulate production of IL‐1ra and IL‐10, a decrease in dysfunctional adipose tissue and improved oxygenation, enhanced innate immune function, and an improved balance of oxylipins.^35^ Aside the direct benefit on the immune system, regular physical activity may also improve the cardiovascular health, blood pressure, and weight control—these help to promote good circulation, a vital process that ensures the cells and other substances of the immune system are freely and adequately transported round the body, especially to a targeted diseased organ.^22^, ^23^ Adequate physical activity, among other things, may also help to reduce and control the feelings of stress and anxiety—two situations that have the potential of modifying a disease state and may also be present in suspected and confirmed COVID‐19 patients.^34^ For these reasons, acute exercise/physical activity is now considered an important factor in strengthening the immune system, especially as it relates to the stimulation of the exchange of leukocytes between the circulation and tissues.^35^

**FIGURE 2:**
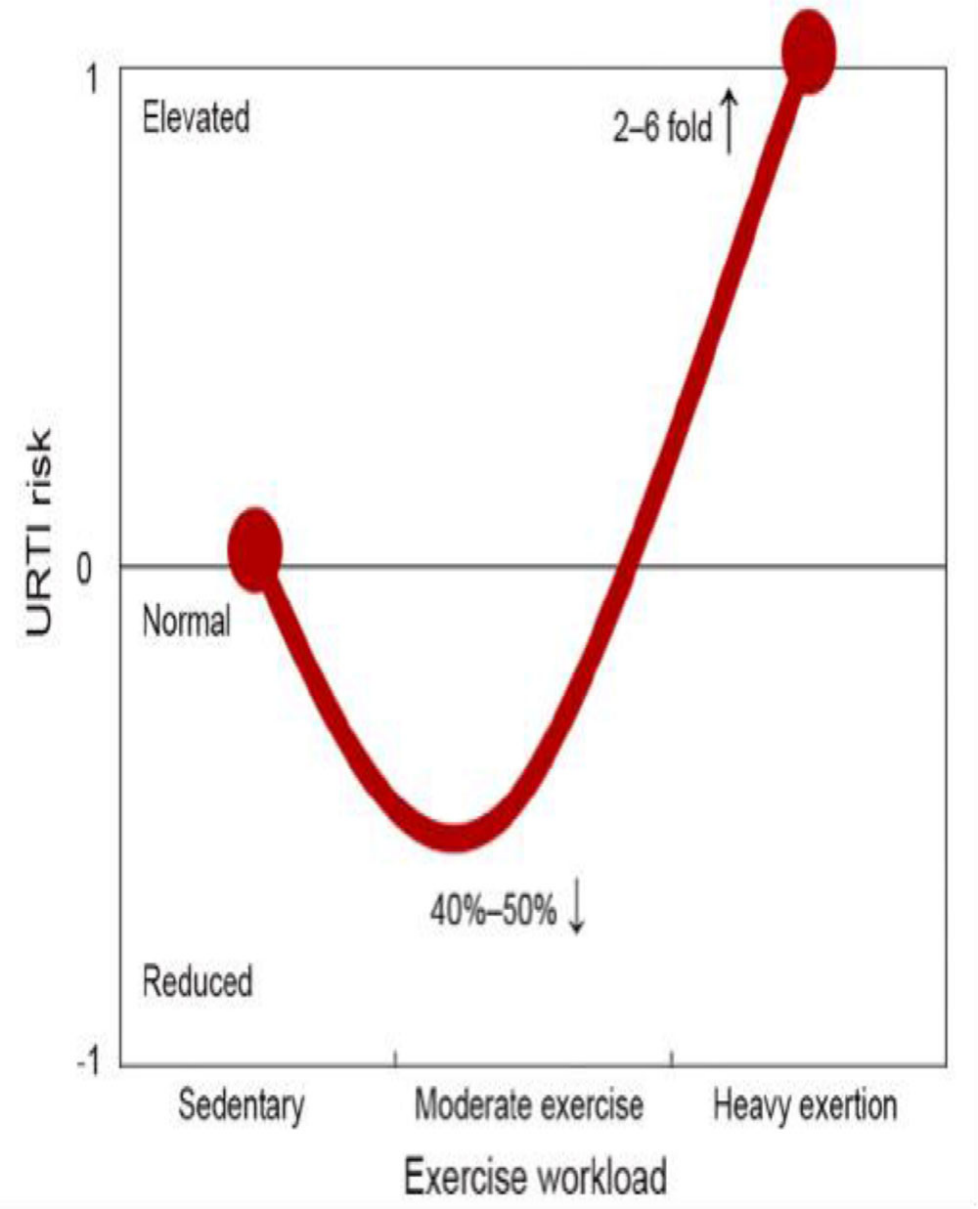
J‐curve model of the relationship between the exercise workload and risk for upper respiratory tract infection. This figure was adapted with permission from Niemanand Wentz^35^

Although stress hormones, which have the potential of suppressing the immune system, and increasing proinflammatory cytokines, are also released during moderate‐intensity exercise, they do not reach significant high clinical levels if the physical activity is done over a relatively short duration of less than 60 min a day.^35^ In contrast, prolonged vigorous‐intensity aerobic exercise (more than 60 min), heavy exertion, or competitive events can result in physiological, metabolic, and psychological stress, and all have been associated with transient immune dysfunction, elevated inflammatory biomarkers, oxidative stress, muscle damage, and an increased risk of URTIs (see Figure 2).^35^ Specifically, such activity can lead to suppression of salivary immunoglobulin A (IgA) output, decreased NK cell lytic activity, reduced T‐ and B‐cell function, and a two‐ to sixfold increased URTI risk up to the second week postactivity.^35^ Accordingly, prolonged vigorous‐intensity physical activity should be discouraged in high‐risk, suspected, and confirmed COVID‐19 cases.

### 4.3 Sleep

Sleep is an essential physiological process that has been found to have significant restorative and regulatory properties on many systems of the body, including the immune system.^36^, ^37^ Many studies have demonstrated that total (especially rapid eye movement) sleep deprivation tends to alter various components of the immune system, such as the percentage of the immune cell subpopulations (eg, CD4+, CD8+, and NK) and cytokine levels (eg, IFN‐g, TNF‐a, and IL‐1).^37^ Both short‐term and long‐term sleep loss have been found to correlate with increased production of many proinflammatory markers such as IL‐6, CRP, and, in some circumstances, TNF, via increased transcriptional activities.^38^ Based on a meta‐analysis, there is an absolute improvement in IL‐6 and CRP levels in response to the elimination of sleep disturbance, which have been found to be equivalent to the absolute positive changes associated with adequate aerobic exercise and healthy dietary interventions.^38^ The vast amount of evidence available goes to emphasize the role of adequate restorative sleep in building and maintaining immunity, especially against infections of viral origin.^31^, ^37^

Specifically, in confirmed COVID‐19 cases, available studies have demonstrated that sleep deprivation tends to increase the CXCL9 levels in the patients—CXCL9 is a substance which increases lymphocytic infiltration and is also implicated in the activation of proinflammatory NLRP3 inflammasome.^11^ Hence, reduced quantity or quality of sleep maybe associated with delay in recovery and increase severity of COVID‐19 cases. Furthermore, adequate sleep also ensures sufficient secretion of melatonin—a molecule which has shown anti‐inflammatory properties and may play a role in reducing coronavirus virulence by reducing oxidative lung injury and inflammatory cell recruitment during the viral infection.^11^ Sleep deprivation is indirectly associated with poor immune response by its tendency to increase higher caloric food craving,^39^ decreased energy stored for physical activity,^40^ and increase substance misuse.^41^

### 4.4 Tobacco use and substance abuse

Although one early study suggested that smoking may be a protective factor in contracting SARS‐CoV‐2, more definitive studies have overturned it.^42^, ^43^ Recent studies have indicated that smokers are more likely to develop severe disease with COVID‐19, compared to nonsmokers.^43^ Available general medical information has also suggested that exposure to tobacco products and other abused substances (like opioids, methamphetamine, and cannabis) tend to cause the body to release massive proinflammatory cells and can cause damages to the body, especially the lung tissues, thereby increasing the risk of contracting viral infections like COVID‐19 and the development of related complications (like pneumonia and acute respiratory distress syndrome).^44^, ^45^, ^46^

Research findings have suggested that smoking tends to have adverse effects on the survival of individuals with infectious diseases of any source.^45^ In addition, findings from other outbreaks caused by coronaviruses from the same family as COVID‐19 suggest that tobacco smoking in particular could, directly or indirectly, contribute to an increased risk of infection, poor prognosis, and/or mortality rate among the infected individuals.^45^ Adult smokers are at increased risk of respiratory infection by several bacterial pathogens, including *Streptococcus pneumoniae*, *Neisseria meningitidis*, *Haemophilus influenzae*, and *Legionella pneumophila*.^47^ It therefore stands to reason that smokers who get infected with the COVID‐19 virus are at increased risk of developing secondary bacterial infections. Furthermore, a peer‐reviewed study in China observed that COVID‐19 patients with the history of smoking had a 14% higher risk of developing COVID‐19 induced pneumonia, while the odds of the disease progressing to stage III (severe stage) and eventually to death were 14 times higher among these sets of patients compared to those who did not smoke.^48^

Likewise, tobacco use has been observed to affect the respiratory system at every level. In particular, it affects the immune function of the nasal cavity by compromising the mucociliary clearance of foreign bodies (like viruses) out of the upper airways.^48^, ^49^ Exposure to tobacco products may also broadly suppress important capacities of the innate immune system, resulting in an ineffective immune response to infections from all sources.^49^ A study involving nasal scrape biopsies from nonsmokers and tobacco users showed extensive immunosuppression at the gene level among the tobacco users. In particular, the expression of more than 60 genes was altered in e‐cigarette users’ alveolar macrophages 2 h after just 20 puffs, including genes involved in inflammation.^49^ Indirectly, tobacco use can increase the risk of contracting COVID‐19 because the users of the tobacco products are more likely to touch their mouth with the fingers.^49^ Also, studies have shown positive correlation between quitting tobacco and better clinical outcome of possible COVID‐19 comorbid conditions (like cardiovascular diseases, diabetes mellitus), which may in turn, lead to better prognosis.^48^

The consumption of other substances like alcohol has also been associated with poor prognosis in the management of viral infections like COVID‐19. Alcohol use, especially heavy use, tends to weaken the immune system, increase the susceptibility to viral infections, and increase the risk of acute respiratory distress syndrome, a possible complication of COVID‐19.^50^ Indirectly, alcohol consumption can increase the risk of suicide in self‐isolating COVID‐19 patients and can also alter peoples’ thoughts, judgment, decision‐making, and behavior—some of which might be critical in COVID‐19 case management.^50^ Alcohol may also affect ability to adhere to the infection prevention and control guidelines on physical distancing, lock down, and self‐isolation. For these reasons, alcohol consumption should be within the recommended safe limit of not more than 14 standard units a week for men and women and drinks spread over 3 days or more if as many as 14 units a week is consumed.^51^ Tobacco products and other harmful substances are best avoided in everyone, but especially in suspected or confirmed COVID‐19 cases.

### 4.5 Stress

Managing a highly contagious disease like COVID‐19 can be a serious challenge to many people. Experiences like observing all the rules of disease management (like self‐isolating and physical distancing), taking medications, dealing with symptoms, making changes to present lifestyle, and the consideration of the possible stigma that may follow the disease are all possible sources of acute and chronic stress in patients with diseases like COVID‐19.^52^ In as much as **s**tress is the body and mind's reaction to everyday tensions, changes, and pressures, too much of this experience can make it more difficult for the body to react appropriately to certain diseases.^52^ There abound scientific evidence that shows direct relationship between chronic stress and the increasing prevalence and progression of some diseases, like viral infections, cardiovascular disease, type 2 diabetes mellitus, asthma, and gastric ulcers.^17^, ^53^ The hormone that is usually released in response to chronic stress, cortisol, has been shown to have the potential to disrupt immune regulation and is specifically associated with increased proinflammatory cytokines such as IL‐6 and suppression of catecholamines and suppressor T‐cells.^11^, ^34^, ^54^ Chronic stress has also been observed to lower the secondary antibody response to infections.^51^ Prolonged stress also tends to result in release of histamines—excess of this can lead to narrowing of the bronchus, and worsen respiratory distress that may result from severe COVID‐19.^54^ Stress can also have an indirect effect on the immune system, as people may use unhealthy behavioral coping strategies to manage their stress, such as heavy alcohol consumption, smoking, and unhealthy diet, which may be counterproductive.^32^, ^49^

A study in which psychological evaluation was done on COVID‐19 patients in isolation wards demonstrated that about 48% of the patients manifested psychological stress during early admission, most of which were from their emotional response to the disease.^29^ A situation which might disrupt their immune response and worsen the disease condition. Consequently, experts have recommended that every confirmed COVID‐19 patient should be evaluated for psychological stress and appropriate measures should be instituted to prevent or manage stress in such patients.^29^

### 4.6 Psychological well‐being and social connectedness

According to **the** relevant literature, there is an association between positive psychological well‐being, social connectedness, and improvement in the human immune response to diseases, especially infectious diseases; while psychological ill‐being is associated with decreased functioning of the immune system.^54^ A study that evaluated the psychological well‐being of COVID‐19 patients observed that many have psychological symptoms—like regret and resentment, loneliness and helplessness, depression, anxiety and phobia, irritation, sleep deprivation, and panic attacks.^29^ Evidence shows that these psychological ill states may disrupt an efficient immune response to the disease condition.^54^ Studies have also shown that isolation and loneliness (for any reason) are associated with increased mortality and morbidity from all causes.^31^ Physical distancing, an essential strategy in the management of suspected or confirmed COVID‐19 case, might become counterproductive if the patients are left isolated or lonely, without any significant social support.^34^ Hence, the maintenance of social connectedness (through different innovative methods), while still observing the physical distancing rules might be more productive in the management of COVID‐19 cases.

A study on Alzheimer's patients observed that, after a period of time, the patients that were provided with companions had stronger immune response compared to those who were not given. This positive immune response was noticed to be associated with positive psychological changes in the patients that connected socially with their companions.^54^ The findings of a meta‐analysis showed that individuals who were described as having negative affective style had a weakened immune system and at risk of contracting infectious diseases compared to the people with positive affective style.^54^ Other studies have demonstrated that people with positive psychological well‐being (PWB) and strong social connections had better immune response to infections as evidenced by changes in serum levels of NK cells, phagocytes, CD3+, CD4+, CD8+, CD4+/CD8+ ratio, and free cortisol.^54^ Religious coping and social support/connection as a measure of boosting PWB have also been shown to be an effective measure in improving the immune response of people living with viral infections like HIV/AIDS.^54^

Affective arousal (which is commonly displayed when people are socially connected) has been found to have positive effect on inflammatory process in the body by modulating the blood level of biomarkers of inflammation—like soluble tumor necrosis factor receptor type II (sTNF‐RII), CRP, and interleukin‐1 receptor antagonist, which in turn can reduce the susceptibility and severity of an infection.^54^ On the flip side, psychological ill‐health, like depression, has been associated with decrease cellular immune response, with consequential delayed recovery from infectious diseases and prolonged wound healing.^51^ Therefore, experts have advocated that the mental and psychological state of the suspected and confirmed COVID‐19 cases should be regularly assessed, monitored, and optimized.^29^, ^54^

## 5 RECOMMENDED LIFESTYLE INTERVENTIONS FOR COVID‐19

Accordingly, see Table 1 for recommended lifestyle intervention guidelines for everyone, but especially for all suspected and confirmed COVID‐19 (stages 1 and 2) cases. These recommendations should form the core of comprehensive supportive care in COVID‐19 management.

**Table 1:** Recommended Lifestyle Interventions for COVID‐19

Lifestyle categories	Recommendations
Nutrition	Fiber‐rich, nutrient‐dense, antioxidant‐rich whole food plant foods such as green leafy vegetables (3‐5 servings/day), fruits (2‐4 servings/day), whole grains, legumes, nuts and seeds Moderate intake of foods with unsaturated fats (like fish, avocado, nuts, olive oil, soy/canola/sunflower/corn oils), white/lean meat and low‐fat dairy products Limit salt consumption, soft drinks, or sodas Avoid foods/snacks containing saturated fats (fatty/red meats, butter, palm/coconut oils, cream, cheese, ghee, lard), trans fats and processed carbohydrates Maintain a healthy weight keeping BMI 18.5‐24.9 kg m^−2^ Aim for optimal hydration: drink 2‐3 L water a day
Physical activity	Engage in moderate‐intensity aerobic physical activity (eg, brisk walk/dance) 30 min‐1 h/day or 150‐300 min per week, plus resistance exercise (eg, press‐ups, situps, and weights) for 1 h, two to three times/week on nonconsecutive days Exercise caution with patients in moderate stage of COVID‐19 who may be breathless on exertion
Sleep	Improving sleep hygiene, aiming for 7‐8 h uninterrupted, restorative sleep every night
Harmful substances	Avoid any form of tobacco products, or any other harmful substances Drink modestly (ensuring alcohol free days) or no alcohol Smoking, vaping, or inhaling any substance can damage the lungs. Make the most of available support for quitting
Stress management	Adopt a variety of measures to keep stress levels low, including favorite music, videos, and movies Talking to a trusted person can help when feeling afraid or sad, avoiding such feelings becoming overwhelming Use reliable sources, such as the World Health Organisation. Minimize anxiety by limiting news, current affairs, and stressful drama. Keep a positive attitude, accepting we cannot control events Consider relaxation techniques such as deep breathing, meditation, Yoga, Tai Chi, and mindfulness, using apps or online resources
Social connectedness	Stay socially connected with loved ones, family and friends by phone, zoom, WhatsApp, and social media if government advice prevents physical meetings Avoid social isolation
Positive psychology	Do activities and direct your thoughts to enhance well‐being and create positive emotions, such as counting blessings, kindness practices, focusing on personal goals/strengths Always find joy, meaning, and purpose, focusing on what makes life worth living Think about the things that call for gratitude and bring happiness Keep your reflections happy and be optimistic about recovery

## 6 CONCLUSION

In order to guarantee the best possible outcome, the management of a devastating disease like COVID‐19 demands a comprehensive and multidisciplinary approach in which all known evidence‐based treatment methods should be adopted. Since the cure of COVID‐19 is not yet known, optimizing the host immune system should be a vital strategy to combat the disease, limit its complications, and reduce mortality. There is some evidence that suggest that the tenacious practice of healthy lifestyle, like healthy diet, regular physical activity, adequate restorative sleep, good stress management, avoidance of tobacco and harmful substances, positive psychological well‐being, and healthy social connections with friends, family, and colleagues can significantly improve the efficacy of the immune response to diverse diseases, especially those of viral origin like COVID‐19.

Lifestyle medicine plays a critical role in primary, secondary, and tertiary prevention of COVID‐19. In primary prevention, pre‐COVID‐19, by adopting healthy lifestyle practices, the host immunity is improved, ready to put up a good fight against the infection, as well as prevent, treat, and where appropriate arrest and reverse the underlying health conditions that place patients at high risk for severe disease and death. Secondary prevention, peri‐COVID‐19, maintaining the core aspects of lifestyle medicine as appropriate in the management of the COVID‐19, and as allowed by infection control guidelines. This will likely control disease progression and severity, shortens recovery time, and improve overall disease prognosis. Tertiary prevention, post‐COVID‐19 recovery, and emerging evidence also support positive impact of healthy lifestyle on the overall physical, psychological, and mental health of recovered COVID‐19 cases.

Lifestyle interventions should form part of the multidisciplinary approach in the management of COVID‐19, alongside other modalities of management, such as pulmonary and cardiac rehabilitation, etc to optimize outcome. We therefore recommend that everyone, susceptible, suspected, and confirmed COVID‐19 cases should be encouraged to adopt healthy lifestyle practices in their daily living. Such measures if practiced consistently should significantly reduce the susceptibility of at‐risk cases, severity, complications, the recovery period, and the fatality rate of the disease. However, it is worth noting that due to some factors like socioeconomic status and environmental conditions, not everybody has the privilege to consistently implement certain lifestyle factors and therefore significant systemic and governmental changes may be required to help those at greatest risk of contracting SARS‐CoV‐2. Since in‐person consultation may be unsafe in this era of COVID‐19, we will also recommend the adoption of virtual group consultations in the management of the asymptomatic and early clinical stages (stages I and II) of COVID‐19.^55^ This method of consultation is also highly recommended for the continued and coordinated care of other patients, especially those with chronic underlying conditions, who are not only at increased risk of contracting the COVID‐19 disease but also have a higher risk of severe disease, with worse outcomes and higher fatality rates. Their normal care may also suffer with healthcare systems all over the world, stretched to their limit in a pandemic such as this. Evidence is also emerging of an association between ethnicity and increased mortality amongst patients with COVID‐19, with the Black, Asian and Ethnic Minorities (BAME) having poorer outcomes.^56^ The comorbid conditions associated with worse COVID‐19 outcomes and the lifestyle factors that contribute to them among the BAME community are often associated with poor socio‐economic status, poor housing, and environmental exposures. These have important implications for policy and it may just be that once these are adjusted for, there may be substantial reduction in risk in these populations. Further research is key to improving the understanding of all the other drivers of poorer outcomes within the BAME community.

## CONFLICT OF INTEREST

The authors declare no conflict of interest.

## AUTHORS CONTRIBUTIONS

IM and ABA conducted the literature review, wrote the manuscript and agreed on the final version of the manuscript. Both authors agree on the order of presentation of the authors.

## Data Availability

Research Data not shared.
